# The Efficacy of Spirulina on Cognitive Function, Psychological and Clinical Indicators in Men Patients Under Methadone Therapy (a Randomized Trial)

**DOI:** 10.1002/fsn3.71521

**Published:** 2026-02-06

**Authors:** Morteza Zamani Asadolah‐poor‐kashi, Peyman Mamsharifi, Freshteh Haerifar, Mehrdad Simani, Amir Ghaderi, Fateme Mehrzad

**Affiliations:** ^1^ Social Determinants of Health (SDH) Research Center Kashan University of Medical Sciences Kashan Iran; ^2^ Student Research Committee Kashan University of Medical Sciences Kashan Iran; ^3^ Department of Psychology Allameh Tabataba‘i University Tehran Iran; ^4^ Clinical Research Development Unit of Shahid Beheshti Hospital Kashan University of Medical Sciences Kashan Iran; ^5^ Clinical Research Development Unit‐Matini/Kargarnejad Hospital Kashan University of Medical Sciences Kashan Iran; ^6^ Department of Psychiatry, School of Medicine Kashan University of Medical Science Kashan Iran

**Keywords:** cognitive function, craving, methadone maintenance therapy, psychological symptoms, spirulina

## Abstract

Patients undergoing methadone maintenance therapy (MMT) often experience psychological issues and clinical dysfunctions, which can negatively impact the effectiveness of the treatment. Addressing these challenges is crucial for improving patient outcomes. This clinical trials aimed to evaluate the effectiveness of Spirulina (SP) supplementation on craving, psychological distress, sexual performance, and cognitive functions in male receiving methadone. In this double‐blind randomized clinical trial, 50 male patients undergoing MMT were randomly assigned to receive either the SP (500 mg twice daily, *n* = 25) or the placebo (*n* = 25) for 12 weeks. Assessments were conducted at baseline and at the end of the intervention. Psychological indicators were measured using the Depression, Anxiety, and Stress Scale‐21 (DASS‐21); sexual function was evaluated with the International Index for Erectile Function (IIEF); drug craving was assessed via the Drug Desire Questionnaire (DDQ); and cognitive performance was measured with the FAS test, Iowa Gambling Task (IGT), and Trail Making Test (TMT). Overall results support that SP supplementation significantly reduced anxiety and stress score compared to the placebo group (*p* < 0.05). However, no significant effects were observed for the depression, sexual function, craving, and cognitive performance measures between the two groups (*p* > 0.05). SP supplementation may help alleviate anxiety and stress in men undergoing MMT, suggesting its potential as a supportive intervention. Further studies with larger participant groups are warranted to confirm these effects and explore broader benefits of SP in this population.

**IRCT Registration:**

IRCT20231101059923N2 (Registration date: 2023‐12‐16).

## Graphical abstract

In male patients receiving methadone maintenance therapy (MMT), Spirulina tablet (500 mg twice daily) for 12 weeks significantly reduced anxiety and stress score compared to the placebo group. However, no significant effects were observed for the depression, sexual function, craving, and cognitive performance measures between the two groups.

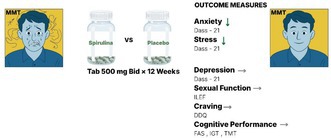

## 1 Introduction

Substance use disorder (SUD) remains a significant public health challenge, affecting millions of individuals across the globe. According to the 2021 World Drug Report by the United Nations Office on Drugs and Crime (UNODC), over 275 million people worldwide engaged in the use of controlled substances in the previous year, with more than 36 million individuals meeting the clinical criteria for SUD (Crime UNOoDa 2021). Among the substances contributing most significantly to the global burden of disease, opioids stand out as the leading cause of drug‐related fatalities, accounting for nearly 69% of all deaths associated with drug use (Lee et al. 2024).

In response to this crisis, methadone maintenance treatment programs (MMTPs) have emerged as a cornerstone in the management of opioid use disorder (OUD) (Ma et al. 2019; Chalabianloo et al. 2024). These programs are pivotal in mitigating opioid cravings and alleviating withdrawal symptoms, offering individuals a structured pathway toward recovery. However, despite its efficacy, methadone therapy is not without its complexities. Patients undergoing treatment often report a range of side effects, including sexual dysfunction, psychological challenges (Anxiety, stress and depression), and broader health concerns (Sanborn et al. 2022; Motazedian et al. 2021; Yee et al. 2014; Cheng et al. 2017). Also, the quality of life of these patients may be affected by various factors, including impaired cognitive functions, craving, social stigma, and financial difficulties (Dalili et al. 2025). Psychologically, it can be triggered by a range of internal and external cues (e.g., stress, physical withdrawal symptoms, and negative emotions) (Ilgen et al. 2008). Furthermore, ongoing struggles with cravings and co‐occurring mental health conditions can undermine the therapeutic benefits of methadone, complicating recovery and diminishing overall quality of life (Wen et al. 2023). These challenges underscore the need for comprehensive, multifaceted approaches to addiction treatment, addressing both the physiological and psychological dimensions of opioid dependence.

Spirulina (Arthrospira platensis) is a nutrient‐dense alga that has garnered recognition for its exceptional protein, vitamin, and mineral content, which exceeds that of many commonly consumed foods like soybeans. Recognized by the United Nations for its nutritional value, Spirulina (SP) is widely used as a dietary supplement due to its powerful antioxidant, antibacterial, antiviral, anti‐inflammatory, and antidiabetic properties (Jung et al. 2019). Studies suggest that SP can enhance the body‘s antioxidant defense systems and has shown potential in improving cognitive function, especially in individuals with neurological disorders (Abd Elkader et al. 2024). Recently, SP supplementation was suggested in subjects with mild to moderately severe depression. This may be due to the beneficial effects of SP intake on mental health disorders in these individuals. This study highlights two favorable benefits of SP supplementation [2 g/day of SP (two 1000 mg) for 8 weeks]: improved sleep latency and quality, and reduced anxiety, depression, and stress scores (Phansuea et al. 2025). Additionally, in an evidence involving multiple sclerosis patients received SP (1 g/day) for 12 weeks exerts anti‐inflammatory effects (IL‐6 and IL‐1β levels), statistically significant also improves physical health, including sexual performance and energy, and potentially mental health compared to placebo (Karimi et al. 2025). In 2025 a systematic review studies suggest that SP may help preserve or improve memory, cognitive performance, and mood by reducing inflammation and oxidative stress (Kumar et al. 2025). In addition to the anti‐inflammatory and antioxidant properties, SP might increase mental health status in MMT patients through many neurobiological mechanisms. SP administration can enhance levels of tryptophan, a precursor of serotonin, thereby promoting central serotonergic activity, which is closely linked to reduced anxiety, improved mood, and increased hopefulness and feelings of well‐being (Demelash 2018). Moreover, SP has been indicated to enhance brain‐derived neurotrophic factor (BDNF) expression, facilitating neuronal repair and neuroplasticity, which might facilitate to better subjective health status and cognitive‐emotional resilience (Trotta et al. 2022; Moradi‐Kor, Ghanbari, et al. 2020). Totally, these mechanisms, likely underlie SP positive effects on quality of life and mental health in the MMT population.

Despite the promising therapeutic effects of SP, no randomized controlled trials (RCTs) to date has directly evaluated SP supplementation in MMTPs. Existing evidence is largely indirect coming from other clinical populations or preclinical models, so the effects of SP on mental health, cognition, and sexual function specifically in MMTPs patients remain unknown. Conducting focused RCTs of SP in this population would address an important knowledge gap and could clarify whether SP is a useful adjunct to improve quality of life, psychological well‐being, cognitive function, and sexual health among individuals on MMTPs. The results of this investigation could provide valuable insights into the potential of SP as a complementary treatment in patients with SUD.

## 2 Methods

### 2.1 Study Population

**FIGURE 1:**
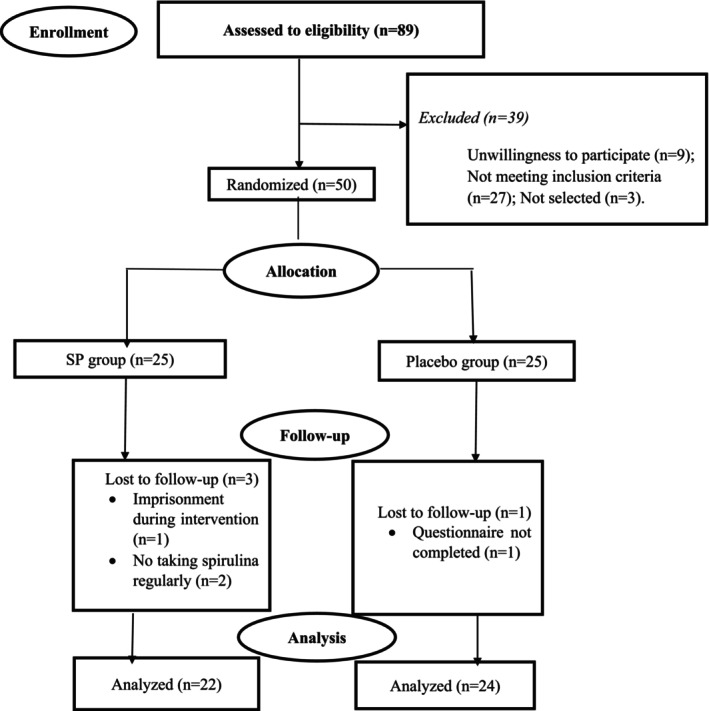
Flowchart of the clinical trial.

The present study employed a randomized, double‐blind, parallel‐group experimental design, including a pretest and posttest. Fifty individuals, aged 30–50 years, who were undergoing MMTPs and seeking treatment at the Mehrparvar Substance Abuse Clinic in Kashan, Iran, participated in the RCTs (Figure 1). This trial was conducted following the ethical guidelines of the Declaration of Helsinki and was approved by the Ethics Committee of Kashan University of Medical Sciences (IR.KAUMS.MEDNT.REC.1402.186). The trial was also registered on the Iranian Clinical Trials website (https://irct.behdasht.gov.ir/trial/73743: IRCT20231101059923N2; Registration date: 2023‐12‐16). Before enrollment, participants were thoroughly informed about the study‘s goals, and they were given 1 week to decide whether to participate. Informed written consent was obtained from all participants, ensuring they fully understood the study's purpose and procedures before joining.

### 2.2 Inclusion and Exclusion Criteria

Inclusion Criteria: (A) Voluntary consent and informed willingness to participate, (B) Positive methadone urine test result, (C) Use of methadone for a minimum of 1 year, (D) Male participants aged between 30 to 50 years.

Exclusion Criteria: (A) Presence of chronic physical conditions, including phenylketonuria, autoimmune diseases, AIDS, hepatitis, liver and kidney disorders, or cardiovascular diseases at the time of enrollment, (B) Inability or failure to participate regularly, (C) Adverse side effects such as insomnia, abdominal discomfort, vomiting, or allergic reactions during the intervention.

### 2.3 Intervention

Participants were randomly assigned to the intervention groups using a block randomization method. First, the SP group was assigned the code “A” and the placebo group was assigned the code “B”. A randomization list was then generated using the website www.sealedenvelope.com/simple‐randomiser/v1/lists, selecting a sample size of 50 participants (with 25 in each group) and applying the permuted block randomization method (block size = 4). Based on this randomization list, subjects were assigned to one of the two groups (A or B). The randomization and group allocation process was concealed from the researchers and participants until the final analysis was completed. A third party, who was not involved in the clinical trial and was unaware of the randomization sequences, assigned the participants to numbered bottles of the corresponding supplements. The intervention group (*n* = 25) received 500 mg of SP capsules twice daily for 12 weeks, the timing and dosage were chosen based on prior evidence to ensure safety and potential therapeutic effects on psychological and clinical parameters (Moradi et al. 2021; Tamtaji et al. 2023). These capsules were provided by Reyhan Naghsh Jahan Pharmaceutical Company, Isfahan, Iran (Product registration code: 9080218987590713). The placebo group (*n* = 25) received two capsules daily, also from Reyhan Naghsh Jahan Company, which contained starch as a control. The placebo capsules were identical in appearance, shape, color, packaging, smell, and taste to the SP capsules. Participants were instructed not to use any additional vitamin or mineral supplements during the 3‐month intervention period and to maintain their regular diet and physical activity levels. Adherence to the intervention was monitored by counting the empty supplement containers at regular intervals. To encourage compliance, all participants received weekly reminders via text‐message on their mobile phones to ensure they took the SP or placebo as prescribed.

### 2.4 Measurements of Outcomes

The primary outcome measures included mental health indicators (depression, anxiety, and stress) and erectile function. Secondary outcome measures focused on craving and cognitive function, assessed using the IGT (Iowa Gambling Task), FAS (FAS‐test), TMT‐A (Trail Making Test Part A), and TMT‐B (Trail Making Test Part B).

### 2.5 Clinical Assessments

Participants enrolled in the MMTPs were asked to complete the study assessments at the clinic (e.g., demographic information). Additionally, all participants were evaluated for psychological indicators, cognitive function parameters, and clinical manifestations (using the DDQ) both before and after the intervention with SP or placebo.

(A) Psychological Measures: The Depression, Anxiety, and Stress Scale‐21 (DASS‐21) are a shortened version of the original DASS, containing 42‐items. The DASS‐21 includes 21 items: seven questions assess depression, seven items measure anxiety, and the remaining items focus on stress. Participants are asked to rate the presence of symptoms experienced in the past week, using a 4‐point Likert scale ranging (0–3). To calculate the final DASS‐21 score, the score for each subscale (depression, anxiety, and stress) is multiplied by two. The Persian version of the DASS‐21 has been validated by Sahebi et al. The internal consistency of the subscales is high, with Cronbach's alpha coefficients of 0.91 (depression), 0.87 (anxiety), and 0.90 (stress) (Lovibond and Lovibond 1995; Yazdi et al. 2019; Sahebi et al. 2005).

(B) Erectile Function: Sexual performance was evaluated using the International Index of Erectile Function (IIEF), a 15‐item questionnaire covering sexual desire, orgasmic, erection, satisfaction, and overall sexual well‐being. Total scores range between 0 and 75, with higher values indicating better sexual functioning. Scores ≤ 25 indicate varying degrees of sexual dysfunction, while 26–30 represent normal function (Rosen et al. 1997).

(C) Drug Craving: The original Drug Desire Questionnaire (DDQ) consists of 13 items designed to assess three key components of drug cravings: desire and intention to use drugs, negative reinforcement, and perceived control. Participants respond to each question on a seven‐point Likert scale, reflecting their feelings or thoughts. Responses are rated on a seven‐point Likert scale from 1 (not at all) to 7 (nearly complete) (Hassani‐Abharian et al. 2016).

(D) Cognitive Function: (1) FAS‐test: The FAS Test, which is part of the Neurosensory Center Comprehensive Examination for Aphasia. The FAS Test evaluates phonemic verbal fluency by requiring participants to generate as many words as possible beginning with the letters F, A, and S within one minute. This task evaluates their ability to access and produce words based on phonemic cues (Crockett 1977). (2) IGT: The Iowa Gambling Task (IGT) is a valuable tool for examining decision‐making processes in individuals. In this task, subjects are presented with four decks of cards. The first two decks offer higher rewards, but they also come with the risk of occasional negative points. In contrast, the other two decks provide smaller rewards but have a significantly lower risk of loss compared to the first two decks. The task determine participants’ ability to make advantageous decisions by weighing the potential rewards against the risks (Turnbull et al. 2014; Businelle et al. 2008).

(3) TMT: The Trail Making Test (TMT) consists of two subtests (e.g., TMT‐A and TMT‐B), designed to assess processing speed and cognitive flexibility. In TMT‐A, individuals are presented with numbers 1 through 25, randomly arranged in circles on a sheet of paper. They are instructed to connect the numbers in ascending order (i.e., 1‐2‐3) using a pen or pencil. In TMT‐B, the test is slightly more complex: the sheet contains both numbers and letters, and individuals must connect them in alternating ascending order (i.e., 1‐A‐2‐B). The performance on each part is usually measured by the time it takes to complete the task, with TMT‐B typically requiring more time than TMT‐A (Stuss et al. 2001; Lamberty et al. 1994).

Together, these validated instruments provided a comprehensive assessment of participants’ psychological well‐being, cognitive performance, and clinical status before and after SP supplementation.

### 2.6 Statistical Analysis

The Kolmogorov–Smirnov test was used to evaluate the normality of the data. Demographic, clinical and general features differences are presented as mean (SD), along with the corresponding frequency distributions. Differences in demographic characteristics between the two groups were done by the two‐sample *t*‐test and Chi‐square test. To evaluate the impact of the treatment intervention on the outcomes of the RCT, the analysis of the covariance (ANCOVA) test was applied, adjusting for baseline values and covariates. Statistical analyses were performed using the SPSS software (version 22, SPSS Inc., Chicago, IL, USA). *p* values < 0.05 were statistically significant.

### 2.7 Sample Size

We were unable to find a similar clinical study examining the effects of SP on cognitive function, psychological, and clinical indicators in individuals undergoing MMTPs, which could guide the sample size calculation based on the primary and secondary outcomes. Therefore, the sample size was determined based on previous research evaluating the effects of SP (Arthrospira platensis) supplementation on blood pressure, anthropometric indices, sleep quality, fatigue, mental health, and quality of life in individuals with ulcerative colitis (Moradi et al. 2021). To detect a mean difference (d) of 5.4 in the absolute change in stress scores as the primary variable between the two groups, with SDs of 7.50 and 5.83 for the SP and placebo groups, respectively, a two‐sided significance level of 0.05, and a power of 80% (α = 0.05, β = 0.20), the required sample size was calculated to be 25 individuals per group under MMTPs.

## 3 Results

A total of 89 individuals under MMTPs were initially screened for eligibility. Based on the study design, 50 patients met the inclusion criteria. These 50 participants were then randomly assigned to receive either SP or a placebo. In the placebo group, one participant did not complete the questionnaire at the end of the treatment period. In the SP group, three participants were excluded for various reasons: two did not adhere to the supplement regimen, and one was incarcerated during the intervention. As a result, 46 patients under MMTPs were included in the final analysis, with 22 in the intervention group and 24 in the placebo group. Figure 1 presents the CONSORT flow diagram for patient enrolment in this RCT.

All participants adhered to the clinical trial protocol and reported no adverse effects from either the SP or placebo supplements. An analysis of demographic and general characteristics revealed that both groups were similar in terms of key variables, including the age at which participants first used drugs, duration of MMTPs, methadone dose, education level, marital status, and other medications used. The mean age in the placebo group was 44.20 ± 4.88 years, while the mean age in the intervention group was 42.81 ± 4.47 years (Table 1).

**TABLE 1:** Demographic and general characteristics of the study patients (*n* = 46)^a^.

Variables	Placebo group (*n* = 24)	SP group (*n* = 22)	*p* ^b^
Age (years)	44.20 ± 4.88	42.81 ± 4.47	0.32
Age of first drug abuse experience (years)	19.50 ± 2.08	19.04 ± 2.12	0.46
Duration MMTPs (years)	11.66 ± 4.07	10.86 ± 3.56	0.48
Methadone dose (mL/d)	27.08 ± 6.90	25.90 ± 8.11	0.59
Education Level (%)			
Elementary	8 (33.3)	6 (27.3)	0.58^c^
Intermediate	9 (37.5)	8 (36.4)	
Diploma	3 (12.5)	6 (27.3)	
University education	4 (16.7)	2 (9.1)	
Marital status (%)			
Single	8 (33.3)	6 (27.3)	0.84^c^
Married	11 (45.8)	10 (45.5)	
Widow/Divorced	5 (20.8)	6 (27.3)	
Other medications used (%)			
None	20 (83.3)	14 (63.6)	0.23^c^
Anti‐depressant	1 (4.2)	4 (18.2)	
Sedative‐Hypnotic	3 (12.5)	4 (18.2)	

^a^ Data are presented as mean ± SD and number (percent).

^b^ Independent *t*‐test.

^c^ Pearson Chi‐square test.

The effect of SP on primary outcomes, including mental health and erectile function, in participants under MMTPs is presented in Table 2. After 12 weeks of SP administration, significant improvements were observed in the anxiety scores (*p* = 0.02). Additionally, significant changes were noted in the stress scores as analyzed by ANCOVA (*p* = 0.03). However, no significant changes were found in depression scores and erectile function indices between the two groups (*p* = 0.06 and *p* = 0.85, respectively).

**TABLE 2:** The effect of SP on primary outcomes in participants under MMTPs (*n* = 46).

Variables	Placebo group (*n* = 24)			SP group (*n* = 22)			β (95% CI)^a^	Effect size	*p* ^b^
	Baseline	End‐of‐trial	Change	Baseline	End‐of‐trial	Change			
Mental health (DASS‐21)									
Depression	20.70 ± 2.72	20.54 ± 2.91	−0.16 ± 1.09	21.09 ± 2.63	20.27 ± 2.58	−0.81 ± 1.36	−0.70 (−1.46, 0.05)	0.081	0.06
Anxiety	17.16 ± 1.90	17.12 ± 1.75	−0.04 ± 1.23	17.09 ± 2.02	16.04 ± 2.31	−1.04 ± 1.32	−0.81 (−1.52, −0.10)	0.118	0.02
Stress	22.62 ± 3.22	22.29 ± 3.45	−0.33 ± 1.52	23.40 ± 4.00	21.81 ± 3.88	−1.59 ± 2.23	−1.22 (−2.36, −0.08)	0.105	0.03
Sexual function (IIEF)	24.83 ± 4.65	25.0 ± 4.61	0.16 ± 1.80	22.81 ± 5.26	22.86 ± 5.17	0.04 ± 1.17	−0.08 (−1.04, 0.86)	0.001	0.85

*Note:* Data are expressed as mean ± SD.

Abbreviations: DASS‐21, depression anxiety and stress scales; IIEF, international index of erectile function.

^a^ β, Difference in the mean outcomes measures among groups; CI, confidence interval.

^b^ Obtained from ANCOVA (Adjusted for baseline values, age, duration of MMTPs, and methadone dose).

The secondary outcomes, such as craving and cognitive function, are reported in Table 3. No significant difference in the DDQ was observed between the two groups (*p* = 0.79). Furthermore, there were no significant differences in the FAS test scores and IGT between the placebo and SP groups when comparing baseline and post‐intervention results (*p* = 0.81 and *p* = 0.30, respectively). Additionally, SP supplementation for 12 weeks did not significantly affect TMT‐A and TMT‐B scores (*p* = 0.29 and *p* = 0.09, respectively).

**TABLE 3:** The effect of SP on secondary outcomes in participants under MMTPs (*n* = 46).

Variables	Placebo group (*n* = 24)			SP group (*n* = 22)			β (95% CI)^a^	Effect size	*p* ^b^
	Baseline	End‐of‐trial	Change	Baseline	End‐of‐trial	Change			
DDQ (craving)	30.58 ± 4.90	30.50 ± 4.76	−0.08 ± 1.05	29.36 ± 4.43	29.04 ± 4.41	−0.31 ± 1.35	−0.08 (−0.76, 0.58)	0.002	0.79
FAS‐test (Numbers total word)	33.41 ± 3.09	33.20 ± 2.57	−0.20 ± 2.02	34.45 ± 2.98	34.13 ± 2.83	−0.31 ± 2.76	0.14 (−1.08, 1.37)	0.001	0.81
IGT (Iowa Gambling Task)	25.08 ± 1.99	25.41 ± 1.81	0.33 ± 1.27	25.72 ± 1.83	25.63 ± 1.83	−0.09 ± 1.15	−0.37 (−1.10, 0.35)	0.026	0.30
TMT (Trail Making Test)									
TMT‐A	30.20 ± 4.07	30.33 ± 4.21	0.12 ± 1.32	30.27 ± 3.16	29.81 ± 3.50	−0.45 ± 1.94	−0.54 (−1.56, 0.48)	0.028	0.29
TMT‐B	84.87 ± 12.04	85.29 ± 12.57	0.41 ± 2.44	83.22 ± 11.64	82.50 ± 11.90	−0.72 ± 2.14	−1.20 (−2.60, 0.19)	0.070	0.09

*Note:* Data are expressed as mean ± SD.

Abbreviations: DDQ craving, desire for drug questionnaire; FAS test, the verbal fluency test; IGT, iowa gambling task; TMT, trail making test.

^a^ β, difference in the mean outcomes measures among groups; CI, confidence interval.

^b^ Obtained from ANCOVA (Adjusted for baseline values, age, duration MMTPs, and methadone dose).

## 4 Discussion

In this clinical trial, we aimed to investigate the effects of SP supplementation on psychological scales, erectile function, cravings, and cognitive performance in patients receiving MMTPs. After 12 weeks of intervention, significant reductions in anxiety and stress were observed in the intervention group, while no significant changes were seen in depression, erectile function, cravings, or cognitive function compared to the placebo group.

These findings are particularly significant given the limited research on the effects of SP in MMTPs patients. SP appears beneficial for reducing stress and anxiety, while the complex and multifactorial nature of SUD, combined with the broad psychological, physiological, and environmental factors, may explain why certain domains such as craving, erectile function and cognitive ability did not significantly improve (Rass et al. 2014). Methadone affects numerous biological systems, mental and physical health, potentially obscuring or interacting with any therapeutic benefits that SP might offer.

Craving in individuals under MMTPs arise from an intricate interaction of environmental, psychological, and neurobiological indicate (Biernacki et al. 2022). In other hand, addictive behaviors are multifaceted and deeply rooted, interventions targeting cravings likely require more comprehensive strategies beyond the anti‐inflammatory and anti‐oxidant effects of SP. However our results shown that SP may alleviate stress and anxiety, its influence on the complex neurobiological mechanisms that underlie craving appears to be minimal. These findings align with earlier reports emphasizing the difficulty of addressing craving and cognitive impairment in individuals maintained on SUD (Lewis et al. 2025; Sorrenti et al. 2021).

Many of evidences have been indicated the relationship between SUD and psychiatric disorders (e.g., depression, stress and anxiety syndromes) (Schuckit 2006). Neuro‐inflammation is an inflammatory response and thought to be connected with a variety of psychiatric disorders (Soytürk et al. 2025). Microalgae including SP exhibit biological activities such as neuroprotective effects, anti‐inflammatory, and antioxidant responses, collectively reducing inflammation‐related depressive behaviors (Bo 2025; Karkos et al. 2011). Our found that MMTPs who were supplemented with SP for 12‐weeks had a significant reduction in anxiety and stress score, but did not significant changes depression symptoms. Prior evidences have suggested that potential anti‐stress and anxiolytic effects of SP via its anti‐oxidant and anti‐inflammatory mechanisms, which help counteract neuro‐inflammation and oxidative stress commonly linked to psychiatric disorders (Sah et al. 2025; Calella et al. 2022). In animal model with exposed to chronic adolescent stress by Moradi‐Kor et al. (Moradi‐Kor, Dadkhah, et al. 2020), *SP platensis* administration, when combined with enriched environments and voluntary exercise, was shown to attenuate oxidative stress, anxiety and depressive‐like symptoms, additionally can protect biochemical defects. This may occur via interactions with the hypothalamic–pituitary–adrenal axis, leading to reduced oxidative damage. In another animal study indicate that SP has adaptogenic activity, and against a several of physiological, biochemical and histological perturbations induced through restraint stress (Juvekar and Nachankar 2003). In addition, improved quality of life was indicated in ulcerative colitis patients following two 500 mg day SP supplementation for 8 weeks, which could be correlated with improved stress status and sleep disturbances, while no significant changes occurred in fatigue, depression and anxiety scores (Moradi et al. 2021). In contrast, a triple‐blind RCT involving patients with hypertension found no significant differences in anxiety levels after 8‐weeks SP supplementation (2 g/day). The researchers suggested that the concurrent emergence of the COVID‐19 pandemic might have influenced outcomes (Far et al. 2022). The inconsistency of the effect of SP in the results of these studies with our study is probably due to differences in population characteristics, duration and dose of intervention, or disease pathology. The evidence indicated that SP microalgae nutrient might be considered as a preventive or therapeutic agent against brain health and reduce stress and anxiety through several mechanisms, including modulating energy metabolism and metabolic function (Sorrenti et al. 2021), upregulate BDNF expression, anti‐inflammatory, antioxidant, and neuroprotective mechanisms (Moradi‐Kor, Ghanbari, et al. 2020), protect the vascular wall of brain vessels and regulate pressure (Sorrenti et al. 2021), modulate cyclooxygenase‐2 (COX‐2) and protect against neurotoxicity (Reddy et al. 2000; Gaurav et al. 2010). Hence, SP alleviating mental health disorder commonly experienced in SUD, further improving patients’ perceived health and quality of life.

Our RCT shown that taking SP for 12‐weeks in patients under MMTPs, it did not influence on sexual function. Prior studies have indicated higher prevalence of sexual dysfunction in patients with opiate dependence under MMTPs (Zafarghandi et al. 2016). Studies in animal models have demonstrated that SP supplementation significantly improves both reproductive and erectile health. In one study, *SP platensis* reduced the negative effects of silver nanoparticles on male rats by increasing testosterone levels, enhancing antioxidant capacity, and reducing malondialdehyde levels. These changes were accompanied by improvements in testicular tissue structure and reduced sperm abnormalities (Moghanlo and Shariatzadeh 2022). Similarly, in another study with rats fed a hypercaloric diet, SP supplementation improved erectile function by enhancing nitric oxide availability, reducing inflammation, and promoting the relaxing effects of acetylcholine. SP also helped restore smooth muscle relaxation in cavernous tissue, decreased reactive oxygen species, and improved overall antioxidant capacity (Diniz et al. 2020). The lack of significant improvements in sexual function in our human study, compared to positive results from animal studies, could be attributed to several factors. Animal models typically have more controlled conditions, such as consistent dosage and treatment duration. At the same time human studies often face greater variability due to factors like individual health, lifestyle, and psychological influences. The divergence between physical sexual function and subjective satisfaction highlights the multidimensional nature of sexual health in SUD (Boggiano et al. 2017). While SP improves physiological capacity, psychological and relational factors may require concurrent targeted interventions (Trotta et al. 2022).

Numerous studies suggest that SP may offer positive effects on cognitive function. In particular, ethanolic extracts of *SP maxima* have been linked to improvements in visual learning, working memory, and vocabulary among older adults with mild cognitive impairment (Choi et al. 2022). Animal studies also show that adding SP to a high‐fat diet can boost spatial memory and learning abilities (Zhou et al. 2021). Clinical research further indicates that SP may enhance cognitive performance, help regulate blood sugar levels, and reduce inflammation in individuals with Alzheimer's disease (Tamtaji et al. 2023). Systematic reviews emphasize the antioxidant and anti‐inflammatory properties of SP, which could play a protective role against neurodegenerative diseases (Kumar et al. 2025). However, the results of this study did not show any significant improvement in cognitive function in the SP group. It's crucial to note that the primary mechanisms through which SP is thought to exert its benefits are its antioxidant and anti‐inflammatory effects. If cognitive decline in the participants is primarily driven by neurotransmitter imbalances, such as dopamine deficiency or disruptions in other neurotransmitter pathways, SP may not directly address these underlying causes (Trotta et al. 2022; Sorrenti et al. 2021). Hence, while SP shows promise in managing inflammation and oxidative stress, its effectiveness in combating cognitive decline tied to neurotransmitter dysfunction remains uncertain.

Our study showed that no significant changes were observed in depression, craving, sexual function, or cognitive performance. This may be due to the short intervention duration, small sample size and the complexity of the conditions being treated. Additionally, relapse, withdrawal symptoms and pain were not assessed, which should be considered in future studies. Previous evidences have linked SP with beneficial microbial shifts, which increase IL‐10 production (Verhoog et al. 2019; Kennedy et al. 2009). Due to the limited budget, this RCT did not include metabolic profiles (Oxidative stress and anti‐inflammatory markers) assessments and gut microbiota analysis, which provided further insight into SP biological effects in MMTPs. Future reports should evaluate microbiome mediated mechanisms and metabolic markers. Furthermore, we did not evaluate the physical activity and dietary intakes in MMTPS; hence, we requested the patients not to change their regular physical activity and dietary intakes.

## 5 Conclusion

In conclusion, this study suggests that SP supplementation may be particularly beneficial for managing anxiety and stress in individuals undergoing MMTPs. However, further research is necessary to explore its potential in improving craving, depression, sexual function, and cognitive health. Future studies should focus on longer treatment durations, larger sample sizes, and a broader range of outcome measures to gain a deeper understanding of SP effects in SUD.

## Author Contributions

**Morteza Zamani Asadolah‐poor‐kashi:** conceptualization (equal), data curation (equal), investigation (equal), methodology (equal), project administration (equal), validation (equal), visualization (equal), writing – original draft (equal), writing – review and editing (equal). **Peyman Mamsharifi:** data curation (equal), investigation (equal), methodology (equal), writing – review and editing (equal). **Freshteh Haerifar:** methodology (equal), validation (equal), visualization (equal), writing – original draft (equal). **Mehrdad Simani:** conceptualization (equal), funding acquisition (equal), methodology (equal), validation (equal), visualization (equal), writing – original draft (equal), writing – review and editing (equal). **Amir Ghaderi:** conceptualization (equal), data curation (equal), funding acquisition (equal), methodology (equal), project administration (equal), resources (equal), software (equal), supervision (equal), validation (equal), visualization (equal), writing – original draft (equal), writing – review and editing (equal). **Fateme Mehrzad:** conceptualization (equal), data curation (equal), funding acquisition (equal), methodology (equal), project administration (equal), resources (equal), software (equal), supervision (equal), validation (equal), visualization (equal), writing – original draft (equal), writing – review and editing (equal).

## Funding

This study was financially supported by Kashan University of Medical Sciences (Clinical Research Development Unit‐Matini/Kargarnejad Hospital), Iran, with a grant code [402110].

## Ethics Statement

The Ethics Committee of Kashan University of Medical Sciences approved the study protocol under the approval number IR.KAUMS.MEDNT.REC.1402.186 and registered at the Iranian Registry of Clinical Trials (https://irct.behdasht.gov.ir/trial/73743: IRCT20231101059923N2) at 16/12/2023. All methods involving human participants in this study adhered to the ethical standards set by institutional and national research committees and were conducted following the principles outlined in the 1964 Helsinki Declaration and its subsequent amendments. All participants provided their informed consent before beginning the study. Each participant received a thorough explanation of the goals, procedures, potential risks, and benefits of the research before giving their agreement. Volunteers were also given the chance to ask any queries they might have had. Participants were informed that there would be no consequences if they chose to withdraw from the study at any time and that participation was completely optional. Confidentiality and privacy were strictly maintained during the entire trial. The anonymized and safely stored identifying personal data was only accessible to the research team. Data were examined in aggregate form to preserve the anonymity of individual responders. Conclusions were presented without any personally identifiable information to adhere to confidentiality rules.

## Consent

The informed consents were completed by all participants.

## Conflicts of Interest

The authors declare no conflicts of interest.

## Data Availability

The datasets analyzed in the current study are available from the corresponding author on reasonable request.
